# Ten tips for successful assessment of risk of bias in randomized trials using the RoB 2 tool: Early lessons from Cochrane

**DOI:** 10.1002/cesm.12031

**Published:** 2023-12-03

**Authors:** Theresa H. M. Moore, Julian P. T. Higgins, Kerry Dwan

**Affiliations:** ^1^ Cochrane Editorial and Methods Department London UK; ^2^ NIHR Applied Research Collaboration West (ARC West) University Hospitals Bristol and Weston NHS Foundation Trust Bristol UK; ^3^ Population Health Sciences, Bristol Medical School University of Bristol Bristol UK; ^4^ Liverpool School of Tropical Medicine Liverpool UK

**Keywords:** Bias, Cochrane, Methodology, Quality assessment, Quality improvement, Randomized controlled trials, Randomized trials, Risk of bias, RoB 2, Systematic reviews

## Abstract

**Introduction:**

RoB 2 is a tool used by systematic reviewers to assess risk of bias in randomized trials. Over a period of 19 months working as editors for Cochrane, we saw many instances where users of RoB 2 frequently applied the tool in ways the developers had not intended, despite availability of detailed guidance, webinars and FAQs.

**Methods:**

In this paper we highlight the ten main issues that we observed, with the aims of optimising the application of the RoB 2 tool, avoiding some of the frequent misapplications of the tool.

**Results:**

Issues noted included failure to state an effect of interest, applying the tool to an entire study rather than to a specific numerical result, omitting key signaling questions and relying on outdated views of causes of bias.

**Conclusion:**

Such omissions and misapplications can lead to overly harsh or lenient assessments of bias with potential to change the confidence we have in an evidence base of randomized trials. We recommend that teams planning to use RoB 2 include at least one member familiar with the RoB 2 detailed guidance and that they use the free resources, such as webinars and FAQs, from the developers of RoB 2 and Cochrane. Our ten tips should be useful to non‐Cochrane systematic reviewers as well as to peer reviewers and editors in Cochrane and other journals.

## 1 INTRODUCTION

The risk of bias 2 (RoB 2) tool was developed for assessing susceptibility to bias in the results of randomized trials (RTs) [1]. In this study, we seek to encourage optimal use of RoB 2 based on our examination of how it was applied during 19 months of editorial peer review of Cochrane systematic reviews. Although the information presented here was drawn from Cochrane reviews, the issues raised are likely to be relevant to authors of all systematic reviews that include RTs and use RoB 2.

After publication of the first Cochrane risk‐of‐bias tool in 2008 [2], researchers found that systematic reviewers frequently applied the tool in ways the developers had not intended. For example, users merged, omitted or added domains, and failed to specify if they applied the tool to specific outcomes [3, 4]. In a survey of 190 users of the original Cochrane risk‐of‐bias tool, just under a third (31%) had modified it, 20% had used it for nonrandomized studies and around half (44%–67%) said they had problems understanding how to complete the domains [5].

After the RoB 2 tool was launched in September 2019, researchers reported that it had not been applied following the guidance [6]. With the knowledge that the new tool differed markedly from the previous version [2], the Cochrane Editorial and Methods Department took steps to avoid the issues of misapplication and misreporting seen for the first Cochrane risk‐of‐bias tool [3, 4, 5]. They planned a phased implementation with coaching for authors and editors and key resources such as a starter pack, frequently asked questions (FAQs), editorial checklists [7, 8, 9], virtual training [10] and monthly methods web clinics [11, 12, 13]. The structure and components of the RoB 2 tool are outlined in Table 1 and described in more detail in the main RoB 2 paper [1] and the detailed guidance at www.riskofbias.info. Differences between the RoB 2 tool and the original risk of bias tool are summarized in the Cochrane guidance to authors [13].

**Table 1:** Structure of the RoB 2 tool for assessing bias in randomized controlled trials.

Feature	About the tool	Comments
Focus of assessment	Results of randomized trials Results of “quasi‐randomized” trials in which allocation was by means other than, but similar to, randomization (e.g., days of the week, birthdate, and so on).	Specific numerical results are assessed. If there is no numerical result for an outcome from a specific study, then there is no need to complete a RoB assessment as it will not contribute to a quantitative synthesis.
Effect of interest	Effect of assignment to intervention or Effect of adhering to a defined intervention	Reviews assessing the effects of an intervention will overwhelmingly be assessing the effect of assignment. The effect of adhering to an intervention can be useful for interventions for adverse events or to take the perspective of the health care user.
Five domains	1. Bias arising from the randomization process 2. Bias due to deviations from intended interventions 3. Bias due to missing outcome data 4. Bias in measurement of the outcome 5. Bias in selection of the reported result	All five domains should be assessed for all trials.
Level of bias	Each domain can be assessed as having: Low risk of bias, Some concerns about risk of bias, or High risk of bias.	The judgment of risk of bias is determined from the answers to a series of “signaling questions” about the trial's conduct and course. An algorithm processes those answers into one of the three judgments.
Signaling questions	Between two and six signaling questions are used to inform the judgments about risk of bias. Answers to signaling questions are Yes/Probably yes No/Probably no No information	The answers to signaling questions are recorded together with a brief reason for the answer.
Algorithm	An algorithm is built into the tool, to enable consistent choice of risk of bias for each domain.	
Overall risk of bias	Overall risk of bias is determined by considering the risks of bias in each of the five domains	Low risk of bias: All domains are Low risk of bias Some concerns: At least one domain is Some concerns and none are High risk of bias High risk of bias: Any single domain is High risk of bias [optional over‐ride] High risk of bias: several domains are Some concerns such that in combination they warrant a judgment of High risk of bias

Abbreviation: RoB 2, risk of bias 2.

Over the period from June 2019 to December 2021 editors in the Cochrane Methods Support Unit (KD and THMM) peer reviewed 144 reviews and protocols that used RoB 2. To ensure transparent and open reporting, Cochrane authors were encouraged to make available a supplemental file containing their consensus agreement for all signaling questions and judgments for all results assessed using RoB 2. These supplemental files were also considered during peer review. During this time we observed that our feedback often repeated comments, indicating there were some common misconceptions in how to apply and present RoB 2.

In this study, we highlight the 10 main issues that we observed, with the aims of optimizing the application of the RoB 2 tool, avoiding some of the frequent misapplications of the tool and demonstrating how to present RoB 2 judgments within a review. These tips are summarized in Table 2 and discussed in the following sections. We hope that our observations will prevent reviewers from reaching judgments that are too harsh or too lenient and help users unfamiliar with the tool become confident to use it.

**Table 2:** Ten tips for assessing risk of bias using RoB 2.

Top 10 tips		Explanation	More information (RoB 2 guidance document) [14]
Aspects to plan in advance			
1	Do plan assessments in advance	State the outcomes (including measures and timepoints) that will be addressed. RoB 2 may be applied separately to all outcomes, or to a subset of outcomes most important to decision makers.	[Section 1]
2	Do state the effect of interest	Choose the effect of interest: either the effect of assignment to intervention or the effect of adhering to intervention. The signaling questions asked in Domain 2 are affected by this decision.	[Section 3]
3	Do pilot the tool to reduce inconsistency in judgments	Develop a review‐specific guidance document to help the team interpret the generic guidance for the specific topic under review. Pilot use of the RoB 2 tool for a few trials and discuss discrepancies to inform this document, to help ensure consistent assessments.	Minozzi et al, 2022 [15]
Factors to consider when applying the tool			
4	Do apply the tool to a specific numerical result and not the whole study	Risk of bias may differ for different outcomes, and even for different results for the same outcome. Avoid attempting to apply the tool to a trial as a whole.	Section 2 & 3
5	Do answer all signaling questions, use the algorithm and provide supporting information for judgments	The RoB 2 tool asks users to complete all signaling questions, provide support for judgments, and use the algorithm to make the bias assessment. Omission of any of these steps can lead to inaccurate assessments (overly harsh or overly lenient) and a lack of transparency.	[Section 1]
Common problems with specific domains			
6	Don't assume baseline imbalance necessarily means bias (Domain 1)	Some degree of baseline imbalance is expected to occur by chance in any randomized trial. It is important to consider whether the imbalance is sufficiently extreme to indicate that something has gone wrong with the randomization process.	[Section 4]
7	Don't assume no blinding means bias (Domains 2 and 4)	Lack of blinding of participants or outcome assessors does not always indicate bias. Randomized trials can be open label yet not troubled by bias. Users should take time to read the guidance and answer all the signaling questions to ensure they consider whether knowing the assignment was likely to lead to bias.	Section 5 & 7
8	Don't assume switching interventions necessarily means bias (Domain 2)	Not all changes in treatment delivered within a trial present a risk of bias. For example, clinicians may change a treatment strategy if a participant's disease progresses. Changes that would occur outside of the trial context, such as due to disease progression, do not introduce bias in the effect of assignment to intervention. Problems arise only if changes in intervention by trial participants happen because of the trial context. We advise authors to check the definition of a “Deviation of intervention” in the detailed guidance and discuss with the review team what would be classed as a deviation from intervention. Draft a RoB 2 consensus document for the review team that includes the definition and examples.	[Section 5]
9	Don't set arbitrary thresholds for missing outcome data (Domain 3)	Avoid setting an arbitrary threshold for assessing the amount of missing outcome data. Ensure that all relevant signaling questions are answered. See the detailed guidance for more information.	Section 7
10	Don't assume the absence of a statistical analysis plan means bias (Domain 5)	A protocol or a trial registration document or a statistical analysis plan can be used to address risk of bias for this domain. It is not necessary to find the formal statistical analysis plan for a randomized trials as frequently these are not available. Some protocols or plans are, unfortunately, registered retrospectively. Therefore it is important to check that registration was before data analysis.	Section 8

*Note*: Section refers to detailed guidance of RoB 2. Higgins et al. Detailed guidance https://www.riskofbias.info/welcome/rob-2-0-tool/current-version-of-rob-2; Minozzi et al. 2022 *Journal of Clinical Epidemiology*, 141, 99–105.

Abbreviation: RoB 2, risk of bias 2.

## 2 ASPECTS TO PLAN IN ADVANCE

### 2.1 Tip 1: Do plan assessments in advance

Outcomes, outcome measurements and timepoints to be assessed for risk of bias should ideally be specified in the protocol. Guidance for the RoB 2 tool recommends that if it is unfeasible to assess risk of bias for all outcomes in a review then a subset of key outcomes, those of most importance to decision makers, may be selected and presented [1, 16]. In many protocols assessed during Cochrane's phased implementation approach, the outcomes for which the review authors planned to assess risk of bias were not stated. In some cases, when outcomes were listed, there was little detail on what time points or measurement methods would be examined. Prespecifying which outcomes will be assessed for risk of bias is helpful because it allows risk‐of‐bias assessments to be performed during initial data extraction. It is also important to be clear what time points and measurement methods are eligible for each synthesis within the review, because this will affect the answers to signaling questions in Domain 5 (bias in the selection of the reported result). This domain is assessed based on the outcome measures of interest to the systematic review and the risk‐of‐bias assessment itself may change depending upon the choice of measurement method and time point chosen by the reviewers, as discussed in Section 8 of the detailed RoB 2 guidance [14].

### 2.2 Tip 2: Do state the effect of interest

A RoB 2 assessment is specific to an effect of interest, which is either the effect of assignment to intervention (the “intention‐to‐treat” effect) or the effect of adhering to an intervention protocol (a “per‐protocol” effect; see Section 1 of the detailed guidance [14]). This effect of interest should be specified in advance, and the choice determines the signaling questions that are asked in Domain 2, “Bias due to deviations from the intended intervention” (see Section 5 of the detailed guidance [14]). We expect the effect of assignment to be used in most instances, although users of the tool may wish to use the latter for outcomes relating to serious adverse events or to reflect the perspective of a health care user, for example when taking part in a mass health screening programmes [1]. In practical terms, choosing the effect of interest and thinking about it early will help to set up risk‐of‐bias assessments, although we observed that many protocols failed to specify it. Being clear which effect of interest is being assessed should reduce the risk of answering the wrong set of signaling questions in Domain 2 (see Sections 1.3 and 5 of the detailed guidance [14]).

### 2.3 Tip 3: Do pilot the tool to reduce inconsistency in judgments

Some risk‐of‐bias assessments we received with completed reviews had inconsistent judgments. For example, we saw instances of users judging a domain as “High risk of bias,” “Some concerns,” or “Low risk of bias” for different studies when the rationale for the different judgments was the same. To address this, we recommend users pilot the RoB 2 tool on a set of results drawn from a range of trial reports, followed by discussion of any discrepancies in their risk‐of‐bias assessments. We also recommend that, where possible, inexperienced users of RoB 2 are paired with more experienced users to do independent assessments. It is useful to draw up a review‐specific guidance document for answering signaling questions that all RoB 2 assessors can refer to. This document can be included in the review as supplementary material [15].

## 3 FACTORS TO CONSIDER WHEN APPLYING THE TOOL

### 3.1 Tip 4: Do apply the tool to a specific numerical result and not the whole study

We know that nearly 43% of published systematic reviews appear to apply RoB 2 to the study as a whole [6]. This is generally inappropriate. Risk of bias may differ for different outcomes from the same study. For example, one team attempted to produce a single risk‐of‐bias judgment for three different outcomes combined: atrial fibrillation, heart rate and adverse effects. However, there were a lot of missing data for adverse effects, which led the team to a “High risk of bias” rating. Thus, the two outcomes atrial fibrillation and heart rate were judged too harshly.

Furthermore, risk of bias may differ for different results, even for the same outcome [1]. For example, a trial report may include a result based on all participants who provided outcome data (i.e., did not drop out) and an alternative analysis in which participants with missing outcomes were addressed through a suitable multiple imputation strategy. These two results may have different risks of bias due to missing outcome data (Domain 3). As another example, a three‐arm trial might contribute two results, one for each of two active interventions against a control group. It may be possible to blind the outcome assessors to one of these interventions but not the other, potentially leading to different risks of bias arising from measurement of the outcome (Domain 4). To ensure risk‐of‐bias assessments are accurate, it is essential that the RoB 2 tool is applied to a specific numerical result for each outcome. Users are asked to specify this numerical result at the outset of the assessment.

### 3.2 Tip 5: Do answer all signaling questions, use the algorithm, and provide supporting information for judgements

We saw frequent examples where users omitted some signaling questions, failed to provide support for their answer, or did not use the in‐built algorithm to generate the risk‐of‐bias judgment. These omissions led to a lack of transparency in how risk‐of‐bias judgments were reached, and in many cases were associated with overly harsh or lenient judgments of risk of bias. A good example is provided by Domain 2, which has two parts and many signaling questions. Part 1 considers deviations from the intended intervention protocol and Part 2 considers the statistical analysis used. We often found users did not complete the second part of the domain and the judgment was based entirely on the first part. Providing a complete assessment is useful to readers of a review since it aids transparency and provides a complete overview of the risk of bias for that result. We encourage users to complete all signaling questions and recommend the use of software implementations of RoB 2, which have the algorithms for reaching risk‐of‐bias judgments built into them reference to RoB 2 Excel tool [17].

## 4 COMMON PROBLEMS WITH SPECIFIC DOMAINS

### 4.1 Tip 6: Don't assume baseline imbalance necessarily means bias

Authors often judged Domain 1 (bias arising from the randomization process) to be “High risk of bias” based only a perception of some baseline imbalance. The wording of the signaling question “Did baseline differences between intervention groups suggest a problem with the randomization process?” is carefully constructed but apparently often ignored. Users frequently answered “Yes” to this question when the baseline imbalance was likely to be due to chance. As an example, one review team awarded a “High risk of bias” rating for this domain on the basis of age and two other baseline characteristics being unbalanced, quoting “*p* < 0.1.” Such a *p* value is insufficiently small to provide an indication of problems with the randomization process, since differences yielding *p* values near to 0.1 are usually highly compatible with chance. The RoB 2 guidance makes it clear that randomization does not achieve perfect balance between intervention groups and that random variation is likely to lead to baseline imbalance. Imbalance is only a problem in the context of RoB 2 if it is so extreme that it indicates that randomization has been compromised. Detailed information on this is provided in Section 4 of the detailed RoB 2 guidance [14].

### 4.2 Tip 7: Don't assume no blinding means bias

We saw many instances of risk‐of‐bias judgments being too harsh when there was a lack of blinding. For example, in Domain 2, lack of blinding during the trial is usually not a problem when the effect of interest is that of assignment to intervention. It was common for a “High risk of bias” judgment to be reached purely on the basis that the participants were aware of intervention received. Further signaling questions were ignored and mitigating effects on bias were not considered. For example, if there were no deviations from intervention, or the deviations were considered not to affect the outcome, judgments of “Low risk of bias” or “Some concerns” could be made. We observed a similar issue for Domain 4 (bias in measurement of the outcome) relating to outcome assessment: it was common for a “High risk of bias” judgment to be reached purely on the basis that outcome assessors were aware of intervention received. Subsequent signaling questions exploring whether this lack of blinding would impact on assessments of the outcome (and hence lead to a risk of bias) were ignored, and so the algorithm was not used.

### 4.3 Tip 8: Don't assume switching interventions necessarily means bias

When assessing Domain 2 for the effect of assignment to intervention, some users misinterpreted the meaning of “deviation from intended intervention.” Some judged a trial result to be at high risk of bias if participants had undergone any changes in their assignment to intervention and they failed to consider whether this change was because of the trial context. For example, it is appropriate, within a clinical trial, for study participants to have their treatment changed if their illness progresses, and such changes are likely to be a part of a trial protocol. This does not represent a deviation from the intended intervention that arose because of the trial context because these changes would happen outside of the trial context as part of the normal care pathway for that patient. In contrast, participants allocated to a control group might seek additional interventions if they realize through the informed consent process that they are not receiving an active intervention, and this would be an example of a deviation that arose because of the trial context. Examples are provided in Box 5 of the detailed guidance [14].

Determining whether deviations from the intended intervention arose because of the trial context requires careful consideration of what those deviations were. Our advice is to discuss potential deviations as a team before the bias assessments are started, in a piloting phase, and decide what will be considered to be a deviation or deviations from intended intervention in trials in their topic area. A guidance document with the group‐made decisions can then be used by all team members assessing bias. This document might itself be piloted on three or four results from different trials and used as an aid to consistency [15].

### 4.4 Tip 9: Don't set arbitrary thresholds for missing outcome data

Risk of bias due to missing outcome data is troublesome to assess for users of the tool. Difficulties with this domain have been noted for the original Cochrane risk of bias tool [5, 18] and by an evaluation of the new Cochrane RoB 2 tool [19]. We observed users struggling with some aspects of their risk‐of‐bias assessments arising from missing outcome data. Some users judged risk of bias on the basis of only two pieces of information: the amount of missing data and whether missing data are balanced between groups. Although we understand the attraction to users of setting a threshold below which they will consider there to be too much missing data, the RoB 2 developers have made it clear that these thresholds are often meaningless because the importance of missing data depends on several factors including the frequency or variability of the outcome and the reasons for the data being missing. The detailed guidance document sets out some approaches to the task of assessing risk of bias resulting from missing outcome data [14]. An important consideration is whether the chance that the outcome is missing depends on the true (unobserved) value of the outcome. This can be a difficult judgment to make and many users did not answer the signaling questions around this. However, it is important to make a judgment about this to understand whether there is a high risk of bias.

### 4.5 Tip 10: Don't assume the absence of a statistical analysis plan means bias

Users often judged a result to be at “High risk of bias” or to have “Some concerns” arising from selection of the reported result based on the single observation that there was no statistical analysis plan. To assess risk of bias for this domain, users are recommended to seek the trialists’ analysis plans. These may be available in formal statistical analysis plans, published protocols or trial registers. Any of these may suffice if sufficiently detailed. It is helpful to compare lists of planned outcomes, outcome measures, timepoints and analyses with the results published in the completed trial report. If the results are in line with the planned analyses and the documentation dated from before unblinded data from the trial were analyzed, then this domain may be judged to be at low risk of bias. Unfortunately, some trial register entries and protocols are created after the start of the trial, and cannot be relied upon to state a priori intentions.

In the absence of detailed documentation of analysis plans, users may use clinical insight to reach a judgment about risk of bias from selective reporting. For example, if the outcome, outcome measure, timepoint and analysis are as one would expect for a particular intervention in a specific clinical area then users may judge the result to be at “low risk of bias.” Examples of outcomes we might expect to be reported include “duration of sleep” for a trial in insomnia, or acute myocardial infarction for a trial in heart disease. The timepoint should also be considered, for example we would expect many outcomes to be measured at the end of the intervention period. For more information see section 8.3.2 of the detailed guidance [14] and a review on bias in the selection of the result [20].

## 5 DISCUSSION

Over a period of 19 months working as editors for Cochrane, we saw many instances where users of the RoB 2 tool had misunderstood how to apply and report risk‐of‐bias assessments in Cochrane reviews and protocols. We report here a distillation of common problems we observed into a series of ten top tips for users of the tool (Table 2).

We observed a combination of misapplications of the tool, which can lead to overly harsh or overly lenient assessments of bias, and the lack of transparency can lead to lack of confidence from review users in the contents of the review. This is especially important as there is an association between poor adherence to RoB 2 guidance and poor review quality [6]. Poor reporting was particularly notable for the effect of interest, for which outcomes were to be assessed and for the rationale for judgments of risk of bias. We suggest that specifying the effect of interest and outcomes in advance in a protocol will help minimize bias, as is the case for all methods for review preparation [21, 22].

Two of the issues we identified have been noted by others, specifically that users often apply the RoB 2 tool to entire studies (rather than specific results), and that users fail to specify what outcomes they plan to assess [6]. Research into reporting of both the original Cochrane risk‐of‐bias tool and the ROBINS‐I tool has found that many review authors do not fully report their judgments or supporting evidence for them, either within the main text of the systematic review or as supplementary information [4, 23]. This is problematic because assessing risk of bias relies on both objective information and subjective considerations. It is important that users of reviews can read how authors reached their decisions so they can decide whether they agree with them [4, 23]. Clear and transparent reporting of research is essential for reproducibility and replication, as is evidenced by the numerous reporting guidelines listed on the EQUATOR (Enhancing the QUAlity and Transparency Of health Research) website and used by researchers and journals [24, 25, 26, 27]. Other work has described the RoB 2 tool as difficult to apply and time‐consuming to use, even by experienced reviewers [15, 28], although has also described similar application times to other tools and more accurate risk‐of‐bias assessments [19].

One reason for the misapplication tool is undoubtedly that assessing risk of bias is a complex process. It requires users to be familiar with the methods of conducting randomized trials, statistical analysis of randomized trials, and the nature and implications of variation in how interventions are implemented in practice. Many of the issues we describe were also reported in an assessment of the original Cochrane risk of bias tool [5]. A reason for the common misapplications of the RoB 2 tool seemed to be that users were unaware of the changing research landscape and the emergence of new understanding regarding causal inference from clinical trials [1, 29]. The lack of take‐up of new research means that understanding of people making bias assessments may have been superseded by developments in the field of bias in randomized trials. For example, in the initial Cochrane risk‐of‐bias tool, no distinction was made between interest in the effect of assignment to intervention and the effect of adhering to intervention, meaning that assessments of risk of bias due to lack of blinding in an open‐label trial were unfocussed. In RoB 2, the distinction allows for open‐label trials to be judged, appropriately, to be at low risk of bias due to deviations from intended interventions.

Another reason for issues with applying the tool might lie with lack of time to for reviewers to learn the new tool. The detailed RoB 2 guidance document is lengthy, and some key aspects might not be immediately obvious to users. For example, the importance of applying the tool to specific results rather than whole randomized trials, of choosing an effect of interest and of completing all of the signaling questions for each domain to inform algorithms represent novel developments that could be overlooked. Piloting the bias tool could also improve consistency in judgments, as has been seen in the use of the ROBINS‐I tool for assessing risk of bias in non‐randomized studies [30]. We recommend that systematic review teams using the RoB 2 tool have at least one member who is fully familiar with the tool and detailed guidance, possibly making use of a series of webinars [10, 14].

**Figure 1:**
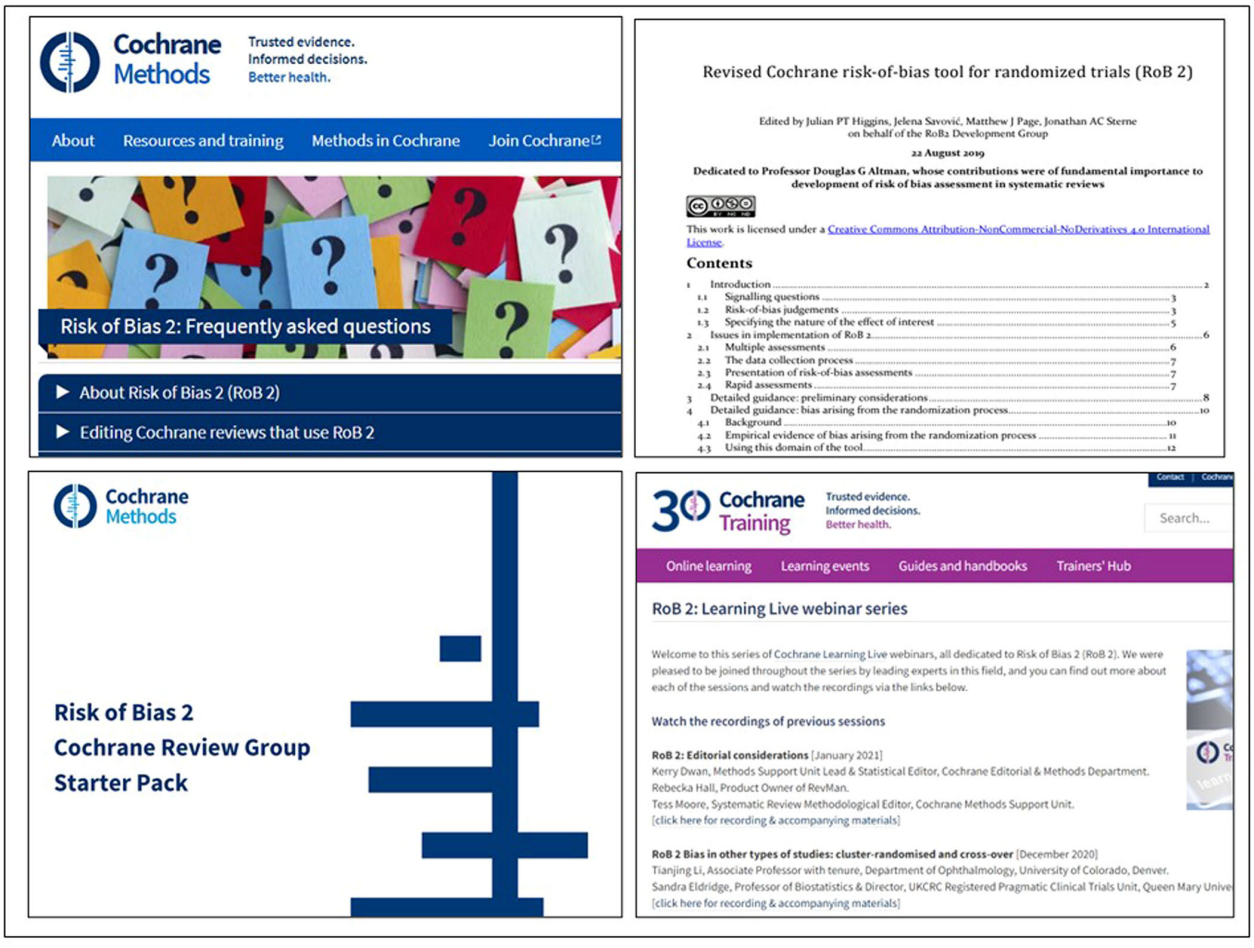
Guidance and training resources for users of risk of bias 2.

There has been little in the way of guidance for reporting risk of bias in systematic reviews [31, 32]. The new PRISMA 2020 checklist elaboration encourages review authors to report the risk of bias judgment for each domain of a tool, along with the rationale [33], but the checklist simply states “Present assessments of risk of bias for each included study” and the full guidance states “briefly summarize the characteristics and risk of bias among studies contributing to the synthesis.” Cochrane have provided additional resources to help familiarize new users to the tool in how to present and report their judgments (Figure 1). The guidance is available for authors of all systematic reviews although some points are specific to Cochrane reviews [8, 10, 12, 13, 34, 35]. Resources are also available for journal editors and peer reviewers [36]. Some of the resources are focussed on Cochrane reviews but could be used for non‐Cochrane reviews.

## 6 CONCLUSIONS

RoB 2 has been used inappropriately in systematic reviews submitted for editorial peer review and without clear reporting of rationales for judgments. Inappropriate use can lead to assessments that may not reflect an appropriate assessment of the risk of bias for an outcome from a randomized trial. Poorly reported risk‐of‐bias assessments may leave users of review without the material to assess their confidence in the review as there is no transparent link between evidence included and conclusions drawn. Resources are available to assist users of the tool and strategies that might help include becoming familiar with the detailed guidance, piloting the tool as a team to reduce inconsistency, engaging with available learning resources. Support for peer reviewers and editors assessing use of RoB 2 is also needed and some guidance for that is also available. Much of the guidance has been developed for Cochrane reviews but could be adapted for use in non‐Cochrane reviews. Timely advice from experienced users of RoB 2 tool at the protocol stage and investment of time in familiarization with the RoB 2 tool by the users can help them to use the tool confidently and assess risk of bias accurately.

Resources are available for users of RoB 2, as well as for editors and peer reviewers who assess systematic reviews that have used RoB 2: Introduction to RoB 2 [37]; Cochrane Starter pack [8]; FAQs [35]; webinar series [10]; detailed RoB 2 guidance [34]; and a Checklist for editors and peer reviewers [36].

## AUTHOR CONTRIBUTIONS

**Theresa H. M. Moore**: Conceptualization; project administration; visualization; writing—original draft; writing—review and editing. **Julian P. T. Higgins**: Conceptualization; writing—original draft; writing—review and editing. **Kerry Dwan**: Conceptualization; writing—original draft; writing—review and editing.

## PEER REVIEW

The peer review history for this article is available at https://www.webofscience.com/api/gateway/wos/peer-review/10.1002/cesm.12031.

## Data Availability

Data sharing is not applicable to this article as no new data were created or analyzed in this study.

## References

[1] Sterne JAC , Savović J , Page MJ , et al. RoB 2: a revised tool for assessing risk of bias in randomised trials. BMJ. 2019;366:l4898. 31462531 10.1136/bmj.l4898

[2] Higgins JPT , Altman DG , Gotzsche PC , et al. The Cochrane Collaboration's tool for assessing risk of bias in randomised trials. BMJ. 2011;343(7829):d5928. 22008217 10.1136/bmj.d5928 PMC3196245

[3] Jørgensen L , Paludan‐Müller AS , Laursen DRT , et al. Evaluation of the Cochrane tool for assessing risk of bias in randomized clinical trials: overview of published comments and analysis of user practice in Cochrane and non‐Cochrane reviews. Syst Rev. 2016;5(1):80. 27160280 10.1186/s13643-016-0259-8 PMC4862216

[4] Puljak L , Ramic I , Naharro CA , et al. Cochrane risk of bias tool was used inadequately in the majority of non‐Cochrane systematic reviews. J Clin Epidemiol. 2020;123:114‐119. 32247026 10.1016/j.jclinepi.2020.03.019

[5] Savović J , Weeks L , Sterne JA , et al. Evaluation of the Cochrane Collaboration's tool for assessing the risk of bias in randomized trials: focus groups, online survey, proposed recommendations and their implementation. Syst Rev. 2014;3(1):37. 24731537 10.1186/2046-4053-3-37 PMC4022341

[6] Minozzi S , Gonzalez‐Lorenzo M , Cinquini M , et al. Adherence of systematic reviews to Cochrane RoB2 guidance was frequently poor: a meta epidemiological study. J Clin Epidemiol. 2022;152:P47‐P55. 10.1016/j.jclinepi.2022.09.003 36156301

[7] Cochrane Methods . Risk of Bias 2 (RoB 2) tool. Cochrane; 2023. Available from https://methods.cochrane.org/risk-bias-2

[8] Cochrane Methods . Risk of bias 2 Cochrane Review Group Starter Pack. Cochrane; 2023. Available from https://methods.cochrane.org/sites/methods.cochrane.org/files/uploads/inline-files/RoB%202_Cochrane%20Starter%20Pack_May2022_modified_080323.pdf

[9] Flemyng E , Moore THM , Boutron I , et al. Using Risk of Bias 2 to assess the risk of bias in results from randomised controlled trials included in systematic reviews: guidance from Cochrane. BMJ Evidence. 2023;28:260‐266. 10.1136/bmjebm-2022-112102 36693715

[10] Cochrane. *RoB 2: Learning Live webinar series*; 2023. Available from https://training.cochrane.org/learning-events/learning-live

[11] Cochrane Methods . Methods support unit web clinic schedule. Cochrane; 2020. Available from https://methods.cochrane.org/about/methods-support-unit/methods-support-unit-web-clinic-schedule

[12] Flemyng E . *Cochrane community blog* [Internet]. Cochrane; 2020. https://community.cochrane.org/news/what-you-need-know-about-risk-bias-2-rob-2-cochrane

[13] Flemyng E , Dwan K , Moore TH , Page MJ , Higgins JP . Risk of bias 2 in Cochrane Reviews: a phased approach for the introduction of new methodology. Cochrane Database Syst Rev. 2020;2020(10):ED000148. 10.1002/14651858.ED000148 PMC10284096 33215687

[14] Higgins J , Savović J , Page MJ , Sterne J . *Revised Cochrane risk of bias tool for randomized trials (RoB 2)*; 2019. https://www.riskofbias.info/welcome/rob-2-0-tool/current-version-of-rob-2

[15] Minozzi S , Dwan K , Borrelli F , Filippini G . Reliability of the revised Cochrane risk‐of‐bias tool for randomised trials (RoB2) improved with the use of implementation instruction. J Clin Epidemiol. 2022;141:99‐105. 34537386 10.1016/j.jclinepi.2021.09.021

[16] Higgins J , Savović J , Page M , Elbers R , Sterne J . Chapter 8: assessing risk of bias in a randomized trial. In: Cochrane Handbook for Systematic Reviews of Interventions Version 62 (updated February 2021) [Internet]. Cochrane; 2021. Available from https://training.cochrane.org/handbook

[17] Risk‐of‐bias.info. An Excel tool to implement RoB 2; 2021. Available from https://www.riskofbias.info/welcome/rob-2-0-tool/current-version-of-rob-2

[18] Babic A , Tokalic R , Cunha JAS , et al. Assessments of attrition bias in Cochrane systematic reviews are highly inconsistent and thus hindering trial comparability. BMC Med Res Methodol. 2019;19(1):76. 30953448 10.1186/s12874-019-0717-9 PMC6451283

[19] Richter B , Hemmingsen B . 2021. *Comparison of the Cochrane risk of bias tool 1 (RoB 1) with the updated Cochrane risk of bias tool 2 (RoB 2)*. Cochrane. https://community.cochrane.org/sites/default/files/uploads/inline-files/RoB1_2_project_220529_BR%20KK%20formatted.pdf

[20] Dwan K , Gamble C , Williamson PR , Kirkham JJ . Systematic review of the empirical evidence of study publication bias and outcome reporting bias ‐ an updated review. PLoS One. 2013;8(7):e66844. 23861749 10.1371/journal.pone.0066844 PMC3702538

[21] Lasserson T , Thomas J , Higgins J . Chapter 1: starting a review. In: Cochrane Handbook for Systematic Reviews of Interventions version 62 (updated February 2021) [Internet]. Cochrane; 2021. Available from https://training.cochrane.org/handbook

[22] Stewart L , Moher D , Shekelle P . Why prospective registration of systematic reviews makes sense. Syst Rev. 2012;1(1):7. 22588008 10.1186/2046-4053-1-7 PMC3369816

[23] Igelström E , Campbell M , Craig P , Katikireddi SV . Cochrane's risk of bias tool for non‐randomized studies (ROBINS‐I) is frequently misapplied: a methodological systematic review. J Clin Epidemiol. 2021;140:22‐32. 34437948 10.1016/j.jclinepi.2021.08.022 PMC8809341

[24] Moher D , Cook DJ , Eastwood S , Olkin I , Rennie D , Stroup DF . Improving the quality of reports of meta‐analyses of randomised controlled trials: the QUOROM statement. Lancet. 1999;354(9193):1896‐1900. 10584742 10.1016/s0140-6736(99)04149-5

[25] Moher D , Liberati A , Tetzlaff J , Altman DG . Preferred reporting items for systematic reviews and meta‐analyses: the PRISMA statement. PLoS Med. 2009;6(7):e1000097. 19621072 10.1371/journal.pmed.1000097 PMC2707599

[26] Equator Network . *EQUATOR (Enhancing the QUAlity and Transparency Of health Research)*, 2021. Available at https://www.equator-network.org/

[27] Page MJ , McKenzie JE , Bossuyt PM , et al. The PRISMA 2020 statement: an updated guideline for reporting systematic reviews. Syst Rev. 2021;10(1):89. 33781348 10.1186/s13643-021-01626-4 PMC8008539

[28] Minozzi S , Cinquini M , Gianola S , Gonzalez‐Lorenzo M , Banzi R . The revised Cochrane risk of bias tool for randomized trials (RoB 2) showed low interrater reliability and challenges in its application. J Clin Epidemiol. 2020;126:37‐44. 32562833 10.1016/j.jclinepi.2020.06.015

[29] Mansournia MA , Higgins JPT , Sterne JAC , Hernán MA . Biases in randomized trials: a conversation between trialists and epidemiologists. Epidemiology. 2017;28(1):54‐59. 27748683 10.1097/EDE.0000000000000564 PMC5130591

[30] Minozzi S , Cinquini M , Gianola S , Castellini G , Gerardi C , Banzi R . Risk of bias in nonrandomized studies of interventions showed low inter‐rater reliability and challenges in its application. J Clin Epidemiol. 2019;112:28‐35. 30981833 10.1016/j.jclinepi.2019.04.001

[31] Whiting P , Savović J , Higgins JPT , et al. ROBIS: a new tool to assess risk of bias in systematic reviews was developed. J Clin Epidemiol. 2016;69:225‐234. 26092286 10.1016/j.jclinepi.2015.06.005 PMC4687950

[32] Shea BJ , Reeves BC , Wells G , et al. AMSTAR 2: a critical appraisal tool for systematic reviews that include randomised or non‐randomised studies of healthcare interventions, or both. BMJ. 2017;358:j4008. 28935701 10.1136/bmj.j4008 PMC5833365

[33] Page MJ , Moher D , Bossuyt PM , et al. PRISMA 2020 explanation and elaboration: updated guidance and exemplars for reporting systematic reviews. BMJ. 2021;372:n160. 33781993 10.1136/bmj.n160 PMC8005925

[34] Anon . RoB 2 tool. A revised Cochrane risk of bias tool for randomized trials, 2021. Available from https://www.riskofbias.info/ 10.1016/j.jclinepi.2020.06.015 32562833

[35] Cochrane Methods . *Risk of Bias 2: Frequently asked questions*. Cochrane; 2023. Available from https://methods.cochrane.org/risk-bias-2-faqs

[36] Cochrane Methods . *Risk of Bias 2: Editorial checklists for RoB 2*. Cochrane; 2023. Available from https://methods.cochrane.org/file/rob-2-editorial-checklists-crgsdocx

[37] Cochrane Methods . *An Introduction to Risk of Bias 2*. Cochrane; 2023. Available from https://methods.cochrane.org/sites/methods.cochrane.org/files/uploads/inline-files/RoB%202%20in%20Cochrane%20Reviews_An%20Introduction_2021_modified_080323.pdf

